# Proposed changes to the reimbursement of pharmaceuticals and medical devices in Poland and their impact on market access and the pharmaceutical industry

**DOI:** 10.1080/20016689.2017.1381544

**Published:** 2017-09-25

**Authors:** Karolina Badora, Aleksandra Caban, Cécile Rémuzat, Claude Dussart, Mondher Toumi

**Affiliations:** ^a^ Creativ-Ceutical Poland, Cracow, Poland; ^b^ Creativ-Ceutical France, Paris, France; ^c^ Laboratoire Parcours Santé Systémique, Université Claude Bernard, Lyon, France; ^d^ Faculté de Médecine Laennec, Université de Lyon, Lyon, France; ^e^ Faculté de Médecine, Laboratoire de Santé, Université de la Méditerranée, Marseille, France

**Keywords:** Pricing and reimbursement, reimbursement policy, drug policy, medical devices, pharmaceuticals, Poland

## Abstract

In Poland, two proposed amendments to the reimbursement act are currently in preparation; these are likely to substantially change the pricing and reimbursement landscape for both drugs and medical devices. Proposed changes include: alignment of medical device reimbursement with that of pharmaceuticals; relaxing the strict reimbursement criteria for ultra-orphan drugs; establishment of an additional funding category for vaccines; introduction of compassionate use, and a simplified reimbursement pathway for well-established off-label indications; appreciation of manufacturers’ innovation and research and development efforts by creating a dedicated innovation budget; introduction of a mechanism preventing excessive parallel import; prolonged duration of reimbursement decisions and reimbursement lists; and increased flexibility in defining drug programmes. Both amendments are still at a draft stage and many aspects of the new regulations remain unclear. Nonetheless, the overall direction of some of the changes is already evident and warrants discussion due to their high expected impact on pharmaceutical and device manufacturers. Here we evaluate the main changes proposed to the reimbursement of drugs, vaccines, and medical devices, and examine the impact they are likely to have on market access and pharmaceutical industry in Poland.

The reimbursement system in Poland is soon likely to change significantly, with two amendments to the reimbursement act currently under discussion. The first amendment relates to the reimbursement of medical devices, aligning it with that of pharmaceuticals [1], while the other one proposes major changes to the overall reimbursement system for drugs and vaccines [2]. Both amendments are still in the relatively early stages of the legislative process, with public consultations completed only in the second half of 2016. The amendment on medical devices was expected to come into force in mid-2017 [3]. Following public consultations, in late April 2017 the revised amendment was subject to cross-departmental discussions and approved by the Permanent Committee of the Government [4], allowing it to be proceeded further. However, as the deadlines for the next legislative steps are not fixed, the actual timeline for implementation is hard to predict. The timeline for the general amendment is also unclear. Major changes to the pricing and reimbursement regulations included in these amendments are listed in Table 1. This article aims to review the key changes proposed to the reimbursement of drugs, vaccines, and medical devices, and assess their potential impact on market access and pharmaceutical industry in Poland.

**Table 1.** A brief comparison of the current and proposed regulations.

	Current regulations	Proposed amendment
Medical devices	Three reimbursement groups: 1. Devices used in the inpatient setting – fully reimbursed (e.g., stents) 2. Devices issued based on special prescription – separate reimbursement rules apply (e.g., hearing aids) 3. Simple devices sold in pharmacies, reimbursed in the same manner as drugs (e.g., wound dressings)	All devices will be reimbursed in the same manner as drugs
Ultra-orphan drugs	Have to meet the same cost-effectiveness criteria as other drugs	Tailored, more relaxed reimbursement criteria, based on price justification rather than cost-effectiveness
Vaccines	NHF reimburses some vaccines in full and others not at all	An additional funding category will be established, with partial NHF funding and patient co-pay
Compassionate use	Not covered by current regulations	Provisions for compassionate use included
Off-label use	Reimbursed in off-label indications specified by the MoH	Reimbursed ‘by default’ in off-labelled indications in which the drug is commonly used, and known to be safe and effective
Innovation and R&D	MoH can take the manufacturer’s R&D activities into account when deciding on reimbursement, but criteria for assessing R&D activities are unclear	Dedicated budget for innovative drugs, R&D-related risk-sharing instruments
Parallel import of drugs	No fixed margin on reimbursable products that are exported, combined with the negotiated manufacturer prices being low compared with many other European countries, result in shortages of some drugs in Polish pharmacies	Fixed margin on exported drugs to limit exporters’ profits and consequently reduce export volume
Drug programmes	Closely linked to reimbursement decisions for included products, so that any changes need approval of all affected manufacturers	Increased flexibility and efficiency of defining drug programs
Duration of reimbursement decision	Fixed at 2, 3 or 5 years, based on how long the drug has been reimbursed in that indication	Set individually during pricing negotiations; up to 5 years
Review of a valid reimbursement decision before its expiry	Currently not possible	The MoH will be able to initiate such review under certain conditions
Updates to the list of reimbursed products	Issued every other month	Issued quarterly

Abbreviations: MoH – Ministry of Health (Polish: Ministerstwo Zdrowia); NHF – National Health Fund (Polish: Narodowy Fundusz Zdrowia); R&D – Research and Development

## Changes to medical device reimbursement

### Definition of medical devices used in Poland

For the purpose of the 2011 Reimbursement Act, medical devices cover medical and *in vitro* diagnostic devices, and supporting equipment [5], which – while not a medical or diagnostic device itself – is necessary for using the device as intended by the manufacturer [6] (e.g., a PC sold together and configured for use with an ultrasound machine or a CT scanner). The proposed amendment leaves this medical device definition unchanged, but adds a concept of a disposable medical device, that is ‘a device intended for single use in only one patient’ [1]. It is worth noting here that Poland uses a broad definition of medical devices. According to the 2010 Medical Devices Act [6], a medical device is

‘any device, tool, software, material or article that, used independently or in combination, including with any accompanying software intended by the device manufacturer to be used for diagnostic or therapeutic purposes and necessary for proper use of the device, is designed by the manufacturer for use in humans in order to:

- diagnose, prevent, monitor, treat, or alleviate the symptoms of a disease,
- diagnose, monitor, treat, alleviate the symptoms, or compensate the consequences of an injury or handicap,
- examine, replace, or modify an anatomical structure or a physiological process,
- prevent conception,

that does not achieve its intended effect through pharmacological, immunological, or metabolic means, but which action may be supported through such means.’

From this definition, it is clear that the proposed changes to medical device reimbursement will affect a wide range of products, including, but not limited to, therapeutic, diagnostic and implantable devices.

### Overview of the current regulations on reimbursement of medical devices

Similar to other European countries, Poland operates a public health insurance system. As of the end of Q1 2017, the national third party payer (National Health Fund [NHF], Polish: Narodowy Fundusz Zdrowia) provided healthcare to over 35.23 million people, including the insured – that is those paying health insurance contributions (25.8 million) and their family members (8.04 million) – and people eligible for publicly funded healthcare for other reasons (1.39 million) [7]. Care is free at point of delivery for those covered by the NHF, although some out-of-pocket payments exist for drugs and medical devices issued in the outpatient sector.

At present, the NHF reimburses three broad categories of medical devices [1]. The first category comprises all types of devices used in the inpatient setting, which are supplied to the patient free of charge (in line with the 2004 Publicly Funded Healthcare Services Act), as are drugs and special nutritional products^1^ ^1^According to the current regulations (the 2011 Reimbursement Act and the 2006 Act on Food and Nutritional Safety [available at: http://isap.sejm.gov.pl/Download?id=WDU20061711225&type=3]), these include infant formulas and dietary products used for special medical purposes (e.g., protein substitutes used in phenylketonuria). The proposed major amendment to the Reimbursement Act aligns the definition of these special nutritional products (termed ‘foodstuffs with special nutritional use’) with the EU definition of foods for special medical purposes, as per article 2g of the Regulation No. 609/2013 of the European Parliament and of the Council of June 2013 [available at: http://eur-lex.europa.eu/legal-content/EN/TXT/PDF/?uri=CELEX:32013R0609&from=EN]. This change is unlikely to have a substantial effect on the types of special nutritional products reimbursed. administered to hospitalised patients [1,8]. This universal full reimbursement includes both devices used within a procedure (e.g., stent placement) and included in its reimbursement, and those used outside medical procedures (e.g., continence pads). Public hospitals are required to conduct tenders for their supplies in line with the regulations on public procurement; price is generally the main selection criterion, although other criteria may also apply, such as quality, delivery timelines, or payment schedule [9]. Tenders usually take place at individual hospital level. Group tenders are not as common in Poland as they are in many other countries, although public hospital managers have been growing more in favour of them lately [10]. Local governments may also support and facilitate group tenders among public hospitals in their area [10]. However, in the private sector, group tenders are commonplace; a single tender may cover supplies for a whole network of private hospitals and outpatient clinics [10].

The remaining two categories encompass devices used in the outpatient setting. Some devices used in the outpatient setting are included on the reimbursement list for drugs [1] – these are available in pharmacies [11] and are generally simple devices that do not require personalisation for each patient, such as dressings and test strips for glucose monitoring. The pricing and reimbursement of this device group is regulated in the same manner as that of drugs [1]. Briefly, that means prices of reimbursed products are fixed and a reimbursement limit is set, defining the maximum amount that can be reimbursed [5]. Varying rates of co-pay apply (0%, small fixed co-pay, 30% and 50%) [5]. Thus, for products priced below the reimbursement limit, patients only cover the co-pay (if applicable), while for products priced above the reimbursement limit patients pay the difference between that limit and the actual price, in addition to the co-pay [5,12]. Further details of the reimbursement regulations are available in the literature [13]. Finally, devices such as prostheses, infusion sets for insulin pumps, glasses and hearing aids, among others, require a special prescription from an appropriate specialist, which has to be approved by the NHF regional office^2^ ^2^In addition to their Warsaw-based headquarters, the NHF has 16 regional offices, one in each administrative province. before the device can be reimbursed [1,14,15]. For this device category, the legislation defines the indications in which the product may be reimbursed, the reimbursement limit (a cap on the reimbursed amount – if the patient chooses a more expensive product, they will need to cover the difference between the limit and the actual price out-of-pocket, in addition to any co-pay), the applicable patient co-pay, how often a new replacement device can be reimbursed, and a separate reimbursement limit for any necessary repairs of the device [15]. The fixed prices and margins that affect drugs and simple devices sold in pharmacies do not apply to this device group, which is freely priced, often leading to excess pricing of reimbursed devices [1].

It is worth noting that, in terms of medical device supply, the boundary between inpatient and outpatient care is not clear-cut. The first category of medical devices comprises all types of devices that are supplied to patients admitted to hospitals and other providers of inpatient care (e.g., psychiatric hospitals, hospices, rehab clinics, etc.) [8,16]. In addition, some devices used in ambulatory/outpatient care (routine or emergency) will also fall into this category if they are necessary for care provision, including devices used for treatment, care, diagnostic and rehabilitation purposes [8,16]. For instance, a patient having a blood test funded by the NHF will not have to pay for the syringes, swabs and storage vials used, even if the test is conducted at their GP clinic or another outpatient clinic (e.g., a diabetes care centre). Similarly, a patient attending an accident and emergency department with a broken leg will not be required to pay for the cast (as having the leg put in a cast would fall under emergency care), but may subsequently be required to purchase an orthosis, which would fall into the third category of devices, i.e., those issued based on a special prescription.

### Proposed changes to medical device reimbursement

In order to improve access and optimise NHF spending on medical devices, the Ministry of Health (MoH) proposed a number of changes to medical devices reimbursement [1]. The project proposes to combine the three different reimbursement categories outlined above using a single approach, whereby the reimbursement of all medical devices would be aligned with that of drugs [1]. Manufacturers will need to submit a pricing and reimbursement application, accompanied by a device quality analysis performed by the Office for Registration of Medicinal Products, Medical Devices and Biocidal Products [1]. Novel devices, for which no equivalent device is available, will be subject to health technology assessment (HTA) and risk-sharing agreements, analogous to those applying to pharmaceuticals [1]. Under this new regulation, following a reimbursement application from the manufacturer, the MoH will approve reimbursement of the device and assign a maximum manufacturer price,^3^ ^3^Article 12 of the Reimbursement Act specifies 13 factors that affect the decision on reimbursement and maximum manufacturer price. Among others, these factors include the opinion of the Economic Commission (which negotiates prices on behalf of the MoH), Polish HTA Agency (AOTMiT) recommendation (if applicable), importance of the condition, clinical efficacy and effectiveness, safety, budget impact and cost-effectiveness. The proposed amendment on reimbursement of medical devices leaves these criteria largely unchanged, but adds the opinion of the Office for Registration of Medicinal Products, Medical Devices and Biocidal Products on the quality of the product as a factor influencing maximum manufacturer price. taking into account the outcome of pricing negotiations with the Economic Commission; devices will also be subject to fixed wholesale and retail margins, and clustered into reference price groups [1]. The MoH will define reference price groups for simple devices sold in pharmacies (second group of devices described above when discussing current regulations) based on reimbursement in the same indication and similar effectiveness [1,5]. For more complex devices issued based on special prescription or used within procedures (groups 1 and 3 above), similar mechanism of action and technical features, adherence to quality standards and cost-effectiveness will also be taken into account when defining reference price groups [1]. A reimbursement limit will be set for each group, and, in case of devices used in the outpatient setting, the amount of co-pay (0, 10, 30 or 50% of the reimbursement limit for devices issued based on special prescription, with no planned changes to the current co-pay levels for simple devices available in pharmacies) will be calculated, depending on patient, disease and device characteristics [1]. In addition to the co-pay, patients will cover any difference between the product price and the reimbursement limit out of pocket [1].

Importantly, devices used as part of a procedure – which are currently included in a bundle payment with the procedure itself – will go through their own, separate pricing and reimbursement process [1]. Patients undergoing a procedure will be able to choose from a range of appropriate devices, opting for a product priced within the limit (which will be the default option, guaranteed to all those insured) or a more expensive device, in which case patients will cover the difference between its price and the reimbursement limit [1]. Patients may also choose an appropriate product that is not reimbursed, covering its full cost [1].

The MoH is hoping that the proposed changes will allow public spending on medical devices to become both more transparent and more efficient [1]. The changes will be introduced gradually to ensure continuity of supply. To allow manufacturers to smoothly transfer their currently reimbursed devices to the new system, the MoH will allow them to do so on preferential terms. When submitting a reimbursement application under the new rules for a device that is already reimbursed under current regulations, manufacturers will not have to pay the application fee or supply the analyses of clinical and pharmacoeconomic data, which would otherwise have to be provided according to the new regulations [1]. The transition to the new system will be regulated through decrees [1]. The MoH will specify groups of devices that may be issued a reimbursement decision, based on a recommendation from the Polish HTA agency, AOTMiT, that will discuss the necessity of reimbursing such a group, its precise scope and the indications/procedures that the devices are used in [1]. Reimbursement decisions will be issued on request of the manufacturer, but such requests may only be filed for device types covered in the decrees [1]. Continence pads may become one of the first medical device groups to fall under the new regulations [17].

### Possible implications of the proposed changes

The attempt to apply a uniform set of rules to the diverse market of medical devices has sparked considerable criticism [17,18]. Manufacturers have raised concerns about the fact that many types of devices – for instance leg prostheses or wheelchairs – vary considerably in their properties and therefore in price [19]. Although devices will be assigned a reference price group based not only on their target indication and mechanism of action, but also on their effectiveness, technology used, adherence to applicable quality standards and cost-effectiveness, for some devices, it may prove difficult to cluster their myriad different types into reference price groups. The reason for this is that devices are usually selected to suit individual patients and their needs (some may even be personalised, e.g., prostheses) and are therefore not as easily comparable as pharmaceuticals [19,20], where generics containing the same active substance may be used interchangeably.

Furthermore, the proposed application fees might be a concern for some, especially smaller, companies [18,21]. Although the amendment itself does not specify the fee amount, a draft decree accompanying the latest amendment version suggests a basic application fee of PLN3,885 (approximately €900) [22,23]. When applying for reimbursement of different device variants (models, versions, sizes, etc.), the fees for consecutive reimbursement applications may be substantially lower at 10–70% of the basic fee, depending on the nature of differences between variants (e.g., a different model will incur a 70% fee, compared with only 10% for a different size) [23]. Nonetheless, the fees may come up to a substantial amount when the manufacturer applies, for example, for reimbursement of a family of related devices.

Finally, introducing out-of-pocket payments for devices used within a procedure provides patients the opportunity to opt in for a higher-quality product, without having to pay for the entire procedure to be conducted in the private sector [21]. However, the proposed amendment requires healthcare providers to allow patients access to only one appropriate device priced within the reimbursement limit, although they can offer more options [1]. As such, device choice for patients who cannot afford out-of-pocket payments may be limited.

More broadly, aligning reimbursement of all groups of medical devices with that of drugs means relying on out-of-pocket payments for products priced above the reimbursement limit. As some patients may be unable to afford out-of-pocket payments for more expensive products [18,20,21], care should be taken to provide patients with a reasonable range of high-quality, affordable devices priced within, or only slightly above, the reimbursement limit.

For manufacturers wanting to access the Polish medical devices market, the proposed changes are indeed revolutionary. Following their introduction, the reimbursement landscape for most devices – with the exception of those already included on the drug reimbursement list – will change completely. While the exact shape of the new regulations remains to be seen, their overall direction is clear, likely bringing substantial challenges – especially for smaller device manufacturers, who will now have to face additional bureaucracy associated with reimbursement applications and, in case of novel devices, HTA.

## Changes to reimbursement policy in general

### Orphan and ultra-orphan drugs

Current regulations apply uniform reimbursement rules to all drugs, regardless of the size of their target patient population. Thus, orphan and ultra-orphan products (targeting diseases that affect <5 in 10,000 people [24], and ≤1 in 50,000 people [2], respectively) are required to demonstrate cost-effectiveness at the current willingness-to-pay (WTP) threshold of three times the gross domestic product (GDP) per capita per quality-adjusted life year (QALY) gained [5] (in 2015, GDP per capita was PLN46,764, or approximately €11,000 [22,25], which would translate into a WTP threshold of approximately PLN140,000 or €33,000 per QALY). This threshold appears rather stringent, given that reimbursement decisions based on clinical value and measures of innovation – rather than a formal cost-effectiveness analysis – may lead to broader coverage for orphan drugs [26], thus improving patient access. The proposed changes exempt ultra-orphan drugs from the aforementioned cost-effectiveness requirements, and focus reimbursement decisions in ultra-orphan indications on factors that may justify the drug’s price, such as clinical effectiveness [27]. However, despite earlier suggestions from MoH officials that the reimbursement changes will apply to both orphan and ultra-orphan products [28], the proposed amendment focuses solely on ultra-rare diseases [29]. A commentary from one of the orphan disease patient organisations interpreted this narrowing of the patient population in focus – from orphan to ultra-orphan indications only – as an attempt to control spending, and criticised the lack of proposed means for increasing the reimbursement level of orphan drugs, given that few orphan products are reimbursed in Poland [29]. At the same time, however, the patient organisation representative noted that the proposed amendment is the first to introduce special provisions for orphan indications, and saw this as a positive change from orphan diseases not being previously considered as a separate entity [29].

### Reimbursement of vaccines

Poland has operated a national vaccination programme since the 1950s [30]. The programme is revised annually and an updated version is published every year by the Chief Sanitary Inspectorate [30]. The National Immunization Technical Advisory Group (NITAG), including the Sanitary-Epidemiology Advisory Board and the Paediatric Group of Experts on Immunisation Program, advises the MoH and recommends vaccines for inclusion in the programme [30,31]. NITAG members include clinicians, epidemiologists, immunologists, paediatricians, public health experts, vaccinology experts and virologists/microbiologists, who may be additionally supported by external experts from the National Institute of Public Health–National Institute of Hygiene (NIPH-NIH), Polish Medical Societies, as well as institutes and universities [31]. The decision to recommend a vaccine for inclusion in the national vaccination programme is evidence-based, factoring in disease burden, efficacy, effectiveness and safety of the vaccine, pharmacoeconomic analysis [31], epidemiological situation in Poland and neighbouring countries, and relevant vaccination policies in other countries [30].

At present, all mandatory vaccines included in the national immunisation programme are fully reimbursed [31]. Other vaccines may be recommended (e.g., flu and human papillomavirus [HPV] vaccines), but are not reimbursed by the NHF [32], which means that – unless they are paid for from other public funds (e.g., the healthcare budgets of local governments) – patients need to cover the entire cost of the vaccination out of pocket [31]. The amendment proposes to introduce partial reimbursement for these vaccines, with patient co-pay likely to be set at 30 or 50% [33]. The proposed reimbursement mechanism is similar to that for drugs, requiring an application from the manufacturer, followed by a HTA and pricing negotiations, before the MoH issues a decision [27]. The MoH is hoping that, by reducing patient costs, the changes will improve access to vaccines that are currently not mandatory according to the national programme [27].

### Compassionate use of medicines

At present, the Polish regulations do not include any guidance on compassionate use of drugs, severely limiting patient access to investigational treatments that show promises in clinical trials but have not yet been granted a marketing authorisation [27]. The amendment introduces the concept of compassionate use in Poland – a change that has been well received by the industry [34]. Eligible patients will be those suffering from a chronic, serious or life-threatening illness, for whom there are no effective approved treatments [2]. The product manufacturer will have to apply for MoH approval of a compassionate use programme; among other items, the application form should include a description of the target patient group together with an estimate of its size, a description of the disease state, with information on the lack of approved products that could be used in this setting, and criteria for patient inclusion and exclusion into the programme [2]. The manufacturer will have a number of obligations linked to the programme; among others, they will be required to monitor patient safety, sign contracts with healthcare providers that would administer the product (if applicable), and assess patients’ medical records to verify eligibility for the programme [2]. Thus, with the introduction of compassionate use programmes, patients who need them most will gain access to novel, investigational therapies, providing additional treatment options where no other therapies exist or are effective. The reimbursement mechanism for compassionate use programmes is, however, unclear at present.

### Off-label use of drugs

Currently, only licensed indications of pharmaceuticals are reimbursed in the outpatient setting (i.e., for prescription-only drugs, devices and special nutritional products sold in pharmacies), although for some drugs additional off-label indications specified by the MoH may also be reimbursed [5,35]. These off-label indications are established through discussions with the relevant Chief Medical Officers^4^ ^4^In Poland, a Chief Medical Officer is the most senior advisor to the government on health-related matters. However, rather than primarily focusing on public health, a Chief Medical Officer is appointed in each medical discipline (e.g., neurology, epidemiology, intensive care, public health, etc.). and AOTMiT [35], allowing the reimbursed indications to be extended beyond those specified in the summary of product characteristics (SPC). For each drug dosage and pharmaceutical form, reimbursed indications (on- and off-label) and prices are available on the reimbursement lists published by the MoH [36], as are the co-pay and reimbursement limit that apply to each indication (see for instance [11] for an example reimbursement list). The amendment proposes extending the ‘default’ reimbursement scenario to off-label indications that are well-grounded in clinical practice, and for which the drug or device is known to be effective and safe [27], which will simplify the reimbursement of established off-label indications. The option to limit reimbursement to a specific condition will, however, remain unchanged, thus separating reimbursement from the licensed indications specified in the SPC [27].

### Focus on innovative drugs, research and development

In line with the 2011 Reimbursement Act [5], the MoH can, at present, when making a decision on reimbursement, take into account the manufacturers’ investments into research and development (R&D) and public health, both in Poland and elsewhere in the European Union (EU) or the European Free Trade Association [27]. However, the current regulations do little to facilitate evaluating R&D activities and their impact on public health in practice [27]. The proposed amendment addresses this issue by linking innovation to pricing and reimbursement decisions. An additional, dedicated reimbursement budget will provide funding for the reimbursement of innovative products (i.e., products for which no equivalent product is reimbursed in the same indication, and those targeting ultra-orphan diseases [2]), especially those developed by manufacturers whose R&D activities have a considerable impact on the Polish economy [27]. This impact will be assessed by the Ministry for Economic Development; their opinion will affect the decision on spending of the investment-based funds and will be taken into account during pricing negotiations [27]. In order for the Ministry of Economic Development to issue an opinion, the manufacturer will have to provide information on revenue, investments made, production volume, net profit or loss, income tax paid, volume of goods and services imported and exported in/out of Poland, spending on R&D activities in Poland, and expenses related to employees and the amount of social and health insurance contributions paid [2]. Details of innovation-based funding mechanisms will be defined in MoH decrees [2], drafts of which are currently not publicly available. At a conference with the Polish pharmaceutical industry, an MoH representative mentioned that the aforementioned opinion from the Ministry for Economic Development will also be taken into account when calculating the incremental cost-effectiveness ratio for innovative products [37]. Furthermore, novel risk-sharing instruments will be introduced, with provision of innovation-based funds contingent on the manufacturer performing R&D activities agreed with the MoH [27]. To control innovation-based spending, the risk-sharing instruments will also state that the manufacturer has to pay back any reimbursement exceeding a specified limit [27].

The MoH is hoping that the aforementioned solutions will promote industry investments into public health through preferential treatment of those manufacturers whose R&D activities are located in Poland [27]. The industry considered these changes to be positive; however, they recognised that little detail on innovation-based reimbursement has been provided thus far [34]. Indeed, no specific criteria for assessing R&D activity have been officially published as of March 2017, and the industry suggestions are also vague, recommending assessment criteria that will promote science- and technology-based economic development [34].

### Risk-sharing instruments and payback applied to health products

The proposed amendment changes the way in which the reimbursement budget is formed to include income from risk-sharing instruments and payback [27]. This can be seen as an attempt to ‘recycle’ public funds spent on reimbursement by re-investing it into the coverage of drugs and medical devices, and has been – unsurprisingly – considered a step in the right direction by the industry [34].

The amendment includes legislation that substantially simplifies payback [27]. At present, the amount of payback is determined through a complex mechanism, triggered by the NHF exceeding its total reimbursement budget [5]. Payback only applies to those products for which the spending on reimbursement has increased since the preceding year, and the amount paid back depends on the reference price group that the product is in [5]. The amendment proposes that payback should be triggered only by the NHF exceeding its budget on outpatient drug reimbursement, without taking into account the total reimbursement budget, or referring to the product’s reference price group [27].

Current exceptions from statutory payback will continue to apply [27]. Thus, products for which risk-sharing instruments are in place – that is, mostly novel, expensive treatments – will be exempt from payback [27]. Changes to the payback mechanism are criticised by domestic manufacturers, who mostly produce – relatively cheap – generics and therefore do not see themselves as really contributing to the NHF exceeding its reimbursement budget [38].

### Parallel import of pharmaceuticals

Drug prices in Poland are among the lowest in Europe. In 2015, a report that compared the prices of innovative oncology drugs in Poland and 12 other European countries (the Czech Republic, France, Germany, the Netherlands, Slovakia, Spain, the UK, Italy, Austria, Hungary, Romania and Switzerland), showed that in Poland the prices of 15 out of 22 drugs were lower than the average price in all the European countries analysed; furthermore, only two drugs exceeded the average price in the 13 countries by more than 10% [39]. Export margins are, at present, not specified in the Reimbursement Act [5], and the fixed wholesale margins are not applied to drugs intended for export, leaving wholesalers free to set their own margins [40]. The combination of free wholesale margins [41] and manufacturer prices being often substantially lower than in Western Europe [42] means that pharmaceuticals – especially cardiovascular drugs, anticoagulants and drugs used to treat asthma – are commonly exported, often leading to shortages of those drugs in Polish pharmacies [42,43]. The amendment proposes that the 5% wholesale margin should apply to all products that are reimbursed, including those intended for export [2]. Thus, the amendment establishes a fixed margin for exporters in an attempt to make pharmaceutical export less profitable and, consequently, limit it [3,34].

### Other aspects of the drug pricing and reimbursement process

The proposed amendment also includes new regulations on a number of other aspects of pricing and reimbursement in Poland. Among other changes, the amendment proposes that the descriptions of drug programmes – specifying the conditions under which costly, novel and otherwise paid for out-of-pocket therapies are fully reimbursed for patients meeting the inclusion criteria – are separated from reimbursement decisions for products included in those programmes [27]. At the moment, the drug programmes and the relevant reimbursement decisions are linked, so that implementing any changes to the programme itself requires the permission of all manufacturers whose products are included in it [27]. The MoH intends on separating the two [27], which will increase the flexibility and efficiency of defining drug programmes – a proposal well-received by the industry [34].

Another proposed change relates to reimbursement decisions themselves, which are currently valid for two, three or five years, depending on how established the drug is in the given indication [5]. This time period is proposed to be extended [27] – at an industry conference, an MoH representative mentioned that the decisions will be valid for up to five years, with the duration determined individually for each product during pricing negotiations [37], rather than being fixed dependent on the time that has passed since the product’s first positive reimbursement decision. However, the amendment also introduces another substantial change, as the MoH will be able to initiate a review of reimbursement conditions before the expiry of an existing reimbursement decision (e.g., within two years, despite a decision being valid for 5 years) [27]. Such review at the discretion of the MoH is not possible under the current regulations [27] and this change is likely to raise concerns amongst the pharmaceutical industry [3,34].

Further, the time period between updates to the list of reimbursed products will also be extended, so that the list is published quarterly [27], rather than every other month, as is currently the case. Finally, the MoH will publish a list of all reimbursement applications received, together with their progress status, in a bid to increase the transparency of the reimbursement process [27].

## Conclusion

Similarly to other healthcare systems in Europe and around the world, the Polish healthcare system faces substantial cost constraints. Patient access to novel medications is often severely restricted and the reimbursement process itself is relatively non-transparent. The MoH is hoping that introducing the proposed changes will resolve some of the issues, optimising the reimbursement process, improving patient access and ensuring public funds are spent more rationally. Indeed, many of the new regulations – such as the introduction of compassionate use schemes, relaxing cost-effectiveness requirements for ultra-orphan drugs, improving the flexibility of defining drug programmes for innovative therapies, reducing the export of reimbursed products, and creating a dedicated innovation-based budget – can be considered important steps along the way towards a better healthcare system attuned to patients’ needs. However, the effective practical implementation of these changes is just as important as the ideas they cover, and that remains largely uncertain, as the MoH has not yet presented many of the draft decrees that would accompany the Reimbursement Act and specify the details of its implementation. Furthermore, issues such as the reimbursement of drugs for orphan diseases are not addressed in the proposed amendments, and some of the new regulations (e.g., the assessment of manufacturers’ R&D activities) appear to require further refinement and consultations with interested stakeholders.

Indeed, while many of the proposed changes have been well received by the pharmaceutical industry, some – including those related to medical devices – have raised considerable criticism. With the reimbursement of medical devices aligned with that of pharmaceuticals, the changes to the reimbursement system are likely to result in closely similar regulations being applied across the market, for pharmaceuticals, medical devices and special nutritional products alike. In the long run, this may potentially simplify access to the Polish market by making the pricing and reimbursement regulations more straightforward to interpret. However, simplifying the regulations alone seems insufficient, despite the apparent high hopes of the MoH regarding the proposed amendment. Although some of the proposed regulations may need to be specified in more detail and/or somewhat revised, there is still room for dialogue between the MoH, manufacturers and patients, given that both amendments are at a relatively early draft stage. It will be interesting to see which of the proposed changes are, in fact, implemented, and what exact shape they take.

## Key highlights

- The proposed changes introduce uniform reimbursement mechanisms for all medical devices, replacing the three distinct reimbursement groups currently in place. The manufacturers applying for reimbursement of their devices will have to face a new, complex procedure, which will also involve HTA of novel devices.
- Rather than being bundled in a single payment, procedures utilising devices (e.g., stent placement) will be reimbursed separately from the device itself, which will allow patients to choose between appropriate devices, opting to make a co-payment if applicable. Thus, patients will no longer have to pay for the procedure to be conducted in the private sector if they wish to use a non-reimbursed device; they will only cover the costs of the device itself.
- The proposed changes relax reimbursement criteria for ultra-orphan drugs, focusing them on price justification rather than economic analysis, which may improve access to these medications.
- Hoping to improve patient access, the amendment introduces partial reimbursement for vaccines that are recommended, but not mandatory. This may lead to wider uptake of these vaccines in Poland.
- The amendment introduces the concept of compassionate use in Poland, allowing patients who have no other treatment options to access innovative products.
- A dedicated innovation-based budget will be formed to fund products for which no equivalent is available or those used in ultra-orphan indications, which may encourage R&D activities within Poland.
- The combination of low prices and unregulated export margins often leads to drug shortages in Polish pharmacies; in response, the amendment proposes a fixed wholesale margin for drugs intended for export, which aims to limit exporters’ profits and consequently reduce pharmaceutical export volume.
